# Supplementary material for: Associations between physical fitness, body composition, and heart rate variability during exercise in older people: exploring mediating factors
Source: PeerJ. 2024 Sep 26;12:e18061. doi: 10.7717/peerj.18061 (PMC11439397; doi:10.7717/peerj.18061)
Supplement: Supplemental Information 1 [file peerj-12-18061-s001.docx]

STROBE Statement—Checklist of items that should be included in reports of ***cross-sectional studies***

|  | Item No | Recommendation |
| --- | --- | --- |
| **Title and abstract** | 1 | (*a*) Indicate the study’s design with a commonly used term in the title or the abstract YES, addressed in the abstract (line: 28) |
|  |  | (*b*) Provide in the abstract an informative and balanced summary of what was done and what was found  YES, addressed in the abstract (lines: 24-40) |
| Introduction | | |
| Background/rationale | 2 | Explain the scientific background and rationale for the investigation being reported  YES, addressed in the introduction (lines: 43-84) |
| Objectives | 3 | State specific objectives, including any prespecified hypotheses  YES, addressed in the introduction (lines: 85-93) |
| Methods | | |
| Study design | 4 | Present key elements of study design early in the paper  YES, addressed in “Aims and Study Design” (lines: 98-100) |
| Setting | 5 | Describe the setting, locations, and relevant dates, including periods of recruitment, exposure, follow-up, and data collection  YES, addressed in “Setting” (lines: 103-107) |
| Participants | 6 | (*a*) Give the eligibility criteria, and the sources and methods of selection of participants  YES, addressed in “Participants” (lines: 111-116) |
| Variables | 7 | Clearly define all outcomes, exposures, predictors, potential confounders, and effect modifiers. Give diagnostic criteria, if applicable  YES, addressed in “instruments” (lines: 153-218) |
| Data sources/ measurement | 8* | For each variable of interest, give sources of data and details of methods of assessment (measurement). Describe comparability of assessment methods if there is more than one group  YES, addressed in “Instruments” (references: 29-43) and in “Procedures” (lines: 129-151) |
| Bias | 9 | Describe any efforts to address potential sources of bias  NO |
| Study size | 10 | Explain how the study size was arrived at  YES, addressed in “Participants” (lines: 121-124) |
| Quantitative variables | 11 | Explain how quantitative variables were handled in the analyses. If applicable, describe which groupings were chosen and why  YES, addressed in “Statistical analysis” (lines: 221-231) |
| Statistical methods | 12 | (a) Describe all statistical methods, including those used to control for confounding  YES, addressed in “Statistical analysis” (lines 221-235) |
|  |  | (*b*) Describe any methods used to examine subgroups and interactions  Addressed in Statistical analysis (lines: 225-231) |
|  |  | (*c*) Explain how missing data were addressed  NO |
|  |  | (*d*) If applicable, describe analytical methods taking account of sampling strategy  Non applicable |
|  |  | (*e*) Describe any sensitivity analyses  NO |
| Results | | |
| Participants | 13* | (a) Report numbers of individuals at each stage of study—eg numbers potentially eligible, examined for eligibility, confirmed eligible, included in the study, completing follow-up, and analysed  YES, figure 1 |
|  |  | (b) Give reasons for non-participation at each stage  YES, figure 1 (line 126) |
|  |  | (c) Consider use of a flow diagram  YES, figure 1 (line 126) |
| Descriptive data | 14* | (a) Give characteristics of study participants (eg demographic, clinical, social) and information on exposures and potential confounders  YES, table 1 (line 244) |
|  |  | (b) Indicate number of participants with missing data for each variable of interest  NO |
| Outcome data | 15* | Report numbers of outcome events or summary measures  YES, figures 2-4 |
| Main results | 16 | (*a*) Give unadjusted estimates and, if applicable, confounder-adjusted estimates and their precision (eg, 95% confidence interval). Make clear which confounders were adjusted for and why they were included  YES, addressed in the results (lines: 285-309) |
|  |  | (*b*) Report category boundaries when continuous variables were categorized  No categorization |
|  |  | (*c*) If relevant, consider translating estimates of relative risk into absolute risk for a meaningful time period  NO |
| Other analyses | 17 | Report other analyses done—eg analyses of subgroups and interactions, and sensitivity analyses  YES, (lines: 285-309) |
| Discussion | | |
| Key results | 18 | Summarise key results with reference to study objectives  YES, Discussion |
| Limitations | 19 | Discuss limitations of the study, taking into account sources of potential bias or imprecision. Discuss both direction and magnitude of any potential bias  YES, Discussion (lines: 383-392) |
| Interpretation | 20 | Give a cautious overall interpretation of results considering objectives, limitations, multiplicity of analyses, results from similar studies, and other relevant evidence  YES, Discussion |
| Generalisability | 21 | Discuss the generalisability (external validity) of the study results  YES, Discussion (e.g. lines: 333-334, 364-366, 277-279) |
| Other information | | |
| Funding | 22 | Give the source of funding and the role of the funders for the present study and, if applicable, for the original study on which the present article is based  YES, Addressed in funding statement |

*Give information separately for exposed and unexposed groups.

**Note:** An Explanation and Elaboration article discusses each checklist item and gives methodological background and published examples of transparent reporting. The STROBE checklist is best used in conjunction with this article (freely available on the Web sites of PLoS Medicine at http://www.plosmedicine.org/, Annals of Internal Medicine at http://www.annals.org/, and Epidemiology at http://www.epidem.com/). Information on the STROBE Initiative is available at www.strobe-statement.org.
