# Supplementary material for: Associations between physical fitness, body composition, and heart rate variability during exercise in older people: exploring mediating factors
Source: PeerJ. 2024 Sep 26;12:e18061. doi: 10.7717/peerj.18061 (PMC11439397; doi:10.7717/peerj.18061)
Supplement: Supplemental Information 3 [file peerj-12-18061-s003.pdf]

# BAI
# INVENTARIO DE ANSIEDAD DE BECK
# BECK ANXIETY INVENTORY

**Normas de aplicación:** Cada ítem del BAI recoge un síntoma de ansiedad y para cada uno de ellos la persona evaluada debe indicar el grado en que se ha visto afectado por el mismo durante la última semana utilizando para ello una escala tipo Likert de cuatro puntos: 0 (Nada en absoluto), 1 (Levemente, no me molestó mucho), 2 (Moderadamente, fue muy desagradable pero podía soportarlo), ó 3 (Gravemente, casi no podía soportarlo).

| **Lista de items** | | | | |
|---|---|---|---|---|
| 1. Hormigueo o entumecimiento. | 0 | 1 | 2 | 3 |
| 2. Sensación de calor. | 0 | 1 | 2 | 3 |
| 3. Temblor de piernas. | 0 | 1 | 2 | 3 |
| 4. Incapacidad de relajarse. | 0 | 1 | 2 | 3 |
| 5. Miedo a que suceda lo peor. | 0 | 1 | 2 | 3 |
| 6. Mareo o aturdimiento. | 0 | 1 | 2 | 3 |
| 7. Palpitaciones o taquicardia. | 0 | 1 | 2 | 3 |
| 8. Sensación de inestabilidad e inseguridad física. | 0 | 1 | 2 | 3 |
| 9. Terrores. | 0 | 1 | 2 | 3 |
| 10. Nerviosismo. | 0 | 1 | 2 | 3 |
| 11. Sensación de ahogo. | 0 | 1 | 2 | 3 |
| 12. Temblores de manos. | 0 | 1 | 2 | 3 |
| 13. Temblor generalizado o estremecimiento. | 0 | 1 | 2 | 3 |
| 14. Miedo a perder el control. | 0 | 1 | 2 | 3 |
| 15. Dificultad para respirar. | 0 | 1 | 2 | 3 |
| 16. Miedo a morirse. | 0 | 1 | 2 | 3 |
| 17. Sobresaltos. | 0 | 1 | 2 | 3 |
| 18. Molestias digestivas o abdominales. | 0 | 1 | 2 | 3 |
| 19. Palidez. | 0 | 1 | 2 | 3 |
| 20. Rubor facial. | 0 | 1 | 2 | 3 |
| 21. Sudoración (no debida al calor). | 0 | 1 | 2 | 3 |
