# Supplementary material for: Associations between physical fitness, body composition, and heart rate variability during exercise in older people: exploring mediating factors
Source: PeerJ. 2024 Sep 26;12:e18061. doi: 10.7717/peerj.18061 (PMC11439397; doi:10.7717/peerj.18061)
Supplement: Supplemental Information 4 [file peerj-12-18061-s004.pdf]

Geriatric Depression Scale - GDS (Enter from CRF)

Número Identificador (PIDN) ______________________

Fecha en la que se realizó la evaluación ______________________

En la siguiente parte de esta entrevista, le haré preguntas sobre sus sentimientos. Para cada pregunta, responda "sí" o "no", dependiendo de cómo se haya sentido la semana pasada, incluyendo hoy.

1. ¿Se siente satisfecho(a) con su vida?	◯ Sí ◯ No ◯ No respondió
2. ¿Ha suspendido muchas de sus actividades e intereses?	◯ Sí ◯ No ◯ No respondió
3. ¿Siente que su vida esta vacía?	◯ Sí ◯ No ◯ No respondió
4.¿Se aburre con frecuencia?	◯ Sí ◯ No ◯ No respondió
5. ¿Está de buen humor la mayor parte del tiempo?	◯ Sí ◯ No ◯ No respondió
6. ¿Tiene miedo de que algo malo le vaya a pasar?	◯ Sí ◯ No ◯ No respondió
7. ¿Se siente feliz la mayor parte del tiempo?	◯ Sí ◯ No ◯ No respondió
8. ¿Se siente a menudo indefenso(a)?	◯ Sí ◯ No ◯ No respondió
9. ¿Prefiere quedarse en la casa, en vez de salir y hacer cosas nuevas?	◯ Sí ◯ No ◯ No respondió
10. ¿Con respecto a su memoria: ¿Siente que tiene más problemas que la mayoría de la gente?	◯ Sí ◯ No ◯ No respondió
11. ¿Piensa que es maravilloso estar vivo(a) en este momento?	◯ Sí ◯ No ◯ No respondió
12. ¿De la forma de cómo se siente en este momento, ¿Se siente inútil?	◯ Sí ◯ No ◯ No respondió
13. ¿Se siente con mucha energía?	◯ Sí ◯ No ◯ No respondió
14. ¿Siente que su situación es irremediable?	◯ Sí ◯ No ◯ No respondió
15. ¿Piensa que la mayoría de las personas están en mejores condiciones que usted?	◯ Sí ◯ No ◯ No respondió

Puntuación total ______________________
(max score = 15, did not complete = 88)
