# Supplementary material for: Associations between physical fitness, body composition, and heart rate variability during exercise in older people: exploring mediating factors
Source: PeerJ. 2024 Sep 26;12:e18061. doi: 10.7717/peerj.18061 (PMC11439397; doi:10.7717/peerj.18061)
Supplement: Supplemental Information 6 [file peerj-12-18061-s006.pdf]

SENIOR FITNESS TEST (SFT)

La batería SFT, diseñada por Rikli y Jones, surgió por la necesidad de crear una herramienta que nos permitiese valorar la condición física de los mayores con seguridad así como de forma práctica.

Muchos de los tests que se han utilizado para valorar la condición física de los mayores son los realizados por jóvenes por lo que no cumplen las normas de seguridad necesarias en este grupo de población y además les suelen resultar algo complejos

Por otro lado existen pruebas específicas para los muy mayores o personas frágiles que no servirían a los mayores sanos ya que estas pruebas valoran únicamente el nivel de independencia.

La SFT tiene unas características que como explicaremos a continuación la hacen más completa y práctica que los test que solían ser utilizados anteriormente.

Cualidades de la SFT (Rikli y Jones, 2001)

La SFT es muy **completa**: Los tests que componen la batería recogen el mayor número de componentes del fitness asociados con la independencia funcional, mientras que otras baterías realizadas para valorar a los mayores se centran únicamente en algún componente concreto.

La SFT puede realizarse en personas con **diferentes edades** entre 60 y 94 años de edad **y niveles de capacidad física y funcional** ya que esta batería cubre un amplio rango de capacidad funcional, desde los más frágiles a los *elite.*

La SFT es de **fácil aplicación** en cuanto al equipamiento y espacio necesarios, por lo que puede realizarse fuera del laboratorio.

La SFT tiene **valores de referencia** expresados en percentiles para cada uno de los test (obtenidos de un amplio estudio realizado a 7000 personas) lo que nos permite comparar los resultados con personas del mismo sexo y edad.

Todas estas cualidades nos permiten utilizar esta batería tanto en el ámbito de investigación como en el de la aplicación práctica. La SFT posee múltiples aplicaciones (Rickli y Jones, 2001):

1. Para **investigar** debido a su gran fiabilidad y validez (especialmente para su uso fuera del laboratorio).
2. Para **evaluar a los individuos** e **identificar factores de riesgo** (gracias a los valores de referencia podemos comparar la capacidad de los mayores evaluados con los rangos normales en individuos de su mismo sexo y edad). También nos permite evaluar en que capacidades físicas obtienen una menor puntuación para poder prevenir la perdida de independencia
3. Para **planificar los programas ya que nos permite** detectar las necesidades individuales consiguiendo de este modo mayor efectividad en los programas**.**
4. Para **educar** a los participantes y alcanzar los objetivos planteados, una cuidadosa interpretación de los resultados obtenidos en los tests ayuda a los participantes a

comprender la relación entre su nivel de fitness y su movilidad funcional. Por otro lado el planteamiento de objetivos aumenta la motivación y ayuda a dar un significado al programa de ejercicio.

5. Para **evaluar los programas** permitiéndonos de este modo valorar la efectividad del programa propuesto.
6. Para **motivar** a los participantes ya que muchos muestran curiosidad por saber cual es su capacidad física y quieren saber que nivel alcanzan respecto a otros individuos con sus mismas características. También las personas competitivas se sienten motivadas intentando alcanzar las puntuaciones más altas de la tabla.
7. Para **mejorar la relación con los estamentos públicos**, midiendo los resultados de un programa podemos documentar la eficacia del mismo y así obtener recursos de estos estamentos para poder llevarlos a cabo en nuestra comunidad.

Procedimientos y consideraciones para la administración de la batería SFT

<u>Pautas a seguir</u>

La SFT fue diseña como una herramienta sencilla y de fácil aplicación, pero debe llevarse a cabo siguiendo unas normas de seguridad y de realización para así conseguir una valoración fiable, segura y eficaz (Rikli y Jones, 2001).

- los examinadores deberán familiarizarse con los procedimientos de cada prueba tanto en su administración como en la recogida de los datos para después adquirir una amplia experiencia en su aplicación antes de realizarlo con los mayores.

- Antes de realizar la batería los participantes deberán completar u documento por escrito de consentimiento donde se les informará sobre los objetivos y los riesgos.

- Tenemos que seleccionar a los participantes ya que algunas personas no podrán realizar los tests: a los que por razones médicas se les contraindica la realización de ejercicio físico, los que han padecido insuficiencia cardiaca congestiva, los que padecen actualmente dolores articulares, dolor en el pecho, vértigos o angina durante el ejercicio o aquellos que tienen una presión sanguínea alta (160/100) no controlada.

- El día anterior a la evaluación los participantes deberán seguir una serie de instrucciones: no realizar actividad física extenuante uno o dos días antes de la valoración, no beber alcohol en exceso 24 horas antes de los tests, comer algo ligero 1 hora antes de las pruebas, llevar ropa y calzado cómodo y seguro, no olvidar en ambientes calurosos gafas de sol y gorra y en ambientes fríos ropa de abrigo, informar al examinador de cualquier circunstancia o condición médica que pudiera afectarle a la hora de realizar las pruebas. También las pruebas de resistencia aeróbica (6 minutos caminando o la de la marcha durante 2 minutos) podríamos realizarlas antes del día de la evaluación para determinar el ritmo a seguir.

- El material necesario para realizar las pruebas debe estar preparado con anterioridad: silla, cronómetro, mancuernas de 5 y 8 libras, escala, cinta adhesiva, un trozo de cuerda o cordón, cinta métrica (5-10 metros), 4 conos, palillos, cinta métrica, regla, contador de pasos, lapiceros, etiquetas de identificación.

- La hoja de registro, donde iremos anotando las puntuaciones debe estar preparada de antemano, a continuación se muestra la hoja utilizada.

**SENIOR FITNESS TEST**			
Día:		H __ M____	Edad _______
Nombre:		Peso________	Altura ______
Tests	1º intento	2º intento	observaciones
1. Sentarse y levantarse de una silla			
2. Flexiones del brazo			
3. 2 minutos marcha			
4. Flexión del tronco en silla			
5. Juntar las manos tras la espalda.			
6. Levantarse, caminar y volverse a sentar.			
*test de caminar 6 minutos. Omitir el test de 2 minutos marcha si se aplica este test.			

(Traducido de Rikli y Jones, 2001)

- El orden de las pruebas es el que se recoge en la ficha anterior pero si realizamos la prueba de 2 minutos marcha deberíamos omitir la de caminar 6 minutos o en el caso de querer hacer las dos pruebas, la de los 6 minutos la haríamos otro día. El peso y la altura puede realizarse en cualquier momento ya que no supone ningún esfuerzo.

- Las condiciones ambientales deben ser seguras y cómodas tanto por la temperatura como la humedad y si aparecen síntomas de sobrecalentamiento o sobreesfuerzo el participante tendrá que parar.

- Si aparecen los siguientes signos que normalmente se relacionan con una situación de esfuerzo excesivo o sobrecalentamiento debemos para inmediatamente: fatiga inusual o dificultad para respirar, vértigo, dolor en el pecho, latidos irregulares del corazón, dolor de cualquier clase, entumecimiento, pérdida de control muscular y de equilibrio, náuseas o vómitos, confusión o desorientación o visión velada.

- Antes de comenzar la valoración debemos tener claro el procedimiento a seguir en caso de emergencia, así como donde está situado el teléfono más cercano o cual es el teléfono de urgencias y en caso de lesión o accidente recoger toda la información relativa al mismo.

<u>Procedimiento para cada prueba</u>

A continuación vamos a realizar una descripción completa de cada uno de los test que componen la batería *Senior Fitness Test* incluyendo el objetivo, el procedimiento, la puntuación y normas de seguridad. Antes de cada prueba el examinador realizará una demostración de forma que el mayor pueda comprenderla y en el caso de las pruebas que requieren cierta velocidad debemos mostrarlas con cierto ritmo para que entiendan que ese es el objetivo de la prueba. (Rickli y Jones, 2001)

CHAIR STAND TEST (Sentarse y levantarse de una silla)
**Objetivo**: Evaluar la fuerza del tren inferior.<br><br>**Procedimiento**:<br>1. El participante comienza sentado en el medio de la silla con la espalda recta, los pies apoyados en el suelo y los brazos cruzados en el pecho.<br>2. Desde esta posición y a la señal de "ya" el participante deberá levantarse completamente y volver a la posición inicial (ver figura 2) el mayor número de veces posible durante 30".<br>3. Tenemos que demostrar el ejercicio primero lentamente para que el participante vea la correcta ejecución del ejercicio y después a mayor velocidad para que así comprenda que el objetivo es hacerlo lo más rápido posible pero con unos limites de seguridad.<br>4. Antes de comenzar el test el participante realizará el ejercicio uno o dos veces para asegurarnos que lo realiza correctamente.<br><br>**Puntuación**:<br>Número total de veces que "se levanta y se sienta" en la silla durante 30".<br>Si al finalizar el ejercicio el participante ha completado la mitad o más, del movimiento (levantarse y sentarse), se contará como completo.<br>Se realiza una sola vez<br><br>**Normas de seguridad**:<br>El respaldo de la silla debe estar apoyado en la pared o que alguien lo sujete de forma estable.<br>Observar si el participante presenta algún problema de equilibrio.<br>Parar el test de forma inmediata si el participante siente dolor.

ARM CURL TEST (Flexiones del brazo)
**Objetivo**: Evaluar la fuerza del tren superior.<br><br>**Procedimiento**:<br>1. El participante comienza sentado en la silla con la espalda recta, los pies apoyados en el suelo y la parte dominante del cuerpo pegado al borde de la silla.<br>2. Cogemos el peso con el lado dominante y lo colocamos en posición perpendicular al suelo, con la palma de la mano orientada hacia el cuerpo y el brazo extendido.<br>3. Desde esta posición levantaremos el peso rotando gradualmente la muñeca (supinación) hasta completar el movimiento de flexión del brazo y quedándose la palma de la mano hacia arriba, el brazo volverá a la posición inicial realizando un movimiento de extensión completa del brazo rotando ahora la muñeca hacia el cuerpo.

4. A la señal de "ya" el participante realizará este movimiento de forma completa el mayor número de veces posible durante 30".
5. Primero lo realizaremos lentamente para que el participante vea la correcta ejecución del ejercicio y después más rápido para mostrar al participante el ritmo de ejecución.
6. Para una correcta ejecución debemos mover únicamente el antebrazo y mantener fijo el brazo (pegar el codo al cuerpo nos puede ayudar a mantener esta posición)

Puntuación:
Número total de veces que "se flexiona y se extiende" el brazo durante 30".
Si al finalizar el ejercicio el participante ha completado la mitad o más, del movimiento (flexión y extensión del brazo), se contará como completa.
Se realiza una sola vez.

Normas de seguridad:
Parar el test si el participante siente dolor.

6-MINUTE WALK TEST (test de caminar 6 minutos)

Objetivo: Evaluación de la resistencia aeróbica.

Preparación: Antes de comenzar la prueba prepararemos el circuito rectangular que tendrá las siguientes medidas: (20 yardas/18,8 m) por (5 yardas/ 4,57m), cada extremo del circuito estará marcado por un cono y cada 5 yardas/ 4,57m lo marcaremos con una línea.

Procedimiento:

1. Se realizará una vez terminadas todas las pruebas.
2. Saldrán de uno en uno cada 10 segundos.
3. A la señal de "ya" el participante caminará tan rápido como le sea posible durante 6 minutos siguiendo el circuito marcado.
4. Para contar el número de vueltas realizado el examinador dará un palillo al participante por cada vuelta realizada o lo marcará en la hoja de registro (~~*IIII*~~ *II*)
5. A los 3 y a los 2 minutos se avisará del tiempo que queda para finalizar la prueba para que los participantes regulen su ritmo de prueba.
6. Cuando pasen los 6 minutos el participante se apartará a la derecha y se colocará en la marca más cercana manteniéndose en movimiento elevando lentamente las piernas de forma alternativa.

Puntuación:
La puntuación se recogerá cuando todos los participantes hayan finalizado la prueba.
Cada palillo o marca en la hoja de registro representa una vuelta (50 yardas/45,7m).
Para calcular la distancia total recorrida multiplicaremos el número de vueltas por 50 yardas o 45,7m.
Se realizará un solo intento el día de la prueba, pero el día anterior todos los participantes practicarán el test para obtener el ritmo de la prueba.

Normas de seguridad:
Seleccionar un área de superficie lisa y que no deslice.
Poner sillas a lo largo del circuito pero fuera del área de circulación (de la prueba).

Aquellos participantes que muestren signos de esfuerzo excesivo interrumpirán el test.

2- MINUTE STEP TEST (2-Minutos Marcha)

Objetivo: Evaluación de la resistencia aeróbica.

Preparación: Antes de comenzar la prueba mediremos la altura a la que tiene que subir la rodilla el participante llevando un cordón desde la cresta ilíaca hasta la mitad de la rotula, después lo mantendremos sujeto desde la cresta ilíaca y lo doblaremos por la mitad marcando así un punto en el medio del muslo que indicará la altura de la rodilla en la marcha. Para visualizar la altura del paso transferiremos la marca del muslo a la pared para que el participante pueda tener una referencia

Procedimiento:
1. A la señal de "ya" el participante comienza a marchar en el sitio el mayor número de veces que le sea posible durante 2 minutos.
2. Aunque las dos rodillas deben llegar a la altura indicada, contabilizaremos el número de veces que la rodilla derecha alcanza la altura fijada.
3. Si el participante no alcanza esta marca le pediremos que reduzca el ritmo para que la prueba sea valida sin detener el tiempo.

Puntuación:
La puntuación corresponderá al número total de pasos completos (dcha.-izq.) que es capaz de realizar en 2 minutos que será el número de veces que la rodilla derecha alcanza la altura fijada.
Se realizará un solo intento el día del test (el día anterior todos los participantes practicarán el test).

Normas de seguridad:
Aquellos participantes que presenten problemas de equilibrio deberían colocarse cerca de una pared o de una silla para poder apoyarse en caso de perdida de equilibrio.
El examinador supervisará a todos los participantes por si existen signos de esfuerzo excesivo.
Al finalizar el test los participantes caminarán despacio durante un minuto.

CHAIR-SIT AND REACH-TEST (Test de flexion del tronco en silla)

Objetivo: Evaluar la flexibilidad del tren inferior (principalmente bíceps femoral)

Procedimiento:
1. El participante se colocará sentado en el borde de la silla (el pliegue entre la parte alta de la pierna y los glúteos debería apoyarse en el borde delantero del asiento).
2. Una pierna estará doblada y con el pie apoyado en el suelo mientras que la otra pierna estará extendida tan recta como sea posible enfrente de la cadera.
3. Con los brazos extendidos las manos juntas y los dedos medios igualados el participante flexionará la cadera lentamente intentando alcanzar los dedos de los pies o sobrepasarlos.
4. Si la pierna extendida comienza a flexionarse el participante volverá hacia la posición

inicial hasta que la pierna vuelva a quedar totalmente extendida.
5. El participante deberá mantener la posición al menos por 2 segundos
6. El participante probará el test con ambas piernas para ver cual es la mejor de las dos (solo se realizará el test final con la mejor de las dos). El participante realizará un breve calentamiento realizando un par de intentos con la pierna preferida.

Puntuación:
El participante realizará dos intentos con la pierna preferida y el examinador registrará los dos resultados rodeando el mejor de ellos en la hoja de registro.
Se mide la distancia desde la punta de los dedos de las manos hasta la parte alta del zapato.
Tocar en la punta del zapato puntuará "Cero"
Si los dedos de las manos no llegan a alcanzar el pie se medirá la distancia en valores negativos (-)
Si los dedos de las manos sobrepasan el pie se registra la distancia en valores positivos (+).

Normas de seguridad:
El respaldo de la silla debe estar apoyado en la pared o que alguien lo sujete de forma estable.
Recordar al participante que exhale el aire lentamente cuando realiza el movimiento de flexión
El participante nunca debe llegar al punto de dolor.
Las personas que padezcan osteoporosis severa o que sientan dolor al realizar este movimiento no deben realizar el test.

BACK SCRATCH TEST (Test de juntar las manos tras la espalda)

Objetivo: Evaluar la flexibilidad del tren superior (principalmente de hombros)

Procedimiento:
1. El participante se colocará de pie con su mano preferida sobre el mismo hombro y con la palma hacia abajo y los dedos extendidos. Desde esta posición llevará la mano hacia la mitad de la espalda tan lejos como sea posible, manteniendo el codo arriba.
2. El otro brazo se colocará en la espalda rodeando la cintura con la palma de la mano hacia arriba y llevándola tan lejos como sea posible, intentando que se toquen los dedos medios de ambas manos.
3. El participante deberá practicar el test para determinar cual es el mejor lado. Podrá realizarlo dos veces antes de comenzar con el test.
4. Debemos comprobar que los dedos medios de una mano están orientados hacia los de la otra lo mejor posible.
5. El examinador podrá orientar los dedos del participante (sin mover sus manos) para una correcta alineación.
6. Los participantes no podrán cogerse los dedos y tirar de ellos.

Puntuación:
El participante realizará dos intentos con el mejor lado antes de comenzar con el test y se anotará en la hoja de registro poniendo un círculo en la mejor de ellas.

Se mide la distancia entre la punta de los dedos medianos de las dos manos.
Si los dedos solo se tocan puntuará "Cero"
Si los dedos de las manos no llegan a tocarse se medirá la distancia en valores negativos (-)
Si los dedos de las manos se solapan se registra la distancia en valores positivos (+).
Siempre se mide la distancia desde la punta de los dedos de una mano a la otra independientemente de la alineación detrás de la espalda.

Normas de seguridad:
Detener el test si el participante siente dolor.
Recordar a los participantes que continúen respirando cuando realicen el estiramiento y eviten movimientos bruscos.

8-FOOT UP-AND-GO TEST (Test de levantarse, caminar y volverse a sentar)

Objetivo: Evaluar la agilidad y el equilibrio dinámico

Preparación: Colocar una silla pegada a la pared y un cono a 8 pies (2,44 metros), medido desde la parte posterior del cono hasta el borde anterior de la silla.

Procedimiento:
1. El participante se sentará en el medio de la silla manteniendo la espalda recta, los pies apoyados en el suelo y las manos sobre sus muslos. Un pie estará ligeramente adelantado respecto al otro y el tronco inclinado ligeramente hacia delante.
2. A la señal de "ya" el participante se levantará y caminará lo más rápido que le sea posible hasta rodear el cono y volver a sentarse.
3. El tiempo comenzará a contar desde el momento que decimos "ya" aunque el participante no haya comenzado a moverse.
4. El tiempo parará cuando el participante se siente en la silla.

Puntuación:
El examinador realizará una demostración de la prueba al participante y el participante lo realizará una vez a modo de prueba.
El test se realizará dos veces y el examinador lo registrará marcando con un círculo la mejor puntuación.

Normas de seguridad:
El examinador se colocará entre el cono y la silla para ayudar al participante en el caso en el que el participante pierda el equilibrio. En las personas más débiles debemos valorar si se levantan y se sientan de forma segura.

HEIGHT AND WEIGHT (Peso y Talla)

Objetivo: Valorar el Índice de Masa Corporal

Procedimiento:
1. Los mayores podrán tener los zapatos puestos mientras realizamos las mediciones de peso y talla realizando posteriormente los ajustes oportunos, ya que si no perderíamos

mucho tiempo.
2. Respecto a la ***Talla***: colocaremos una cinta métrica pegada a la pared en posición vertical a 20 pulgadas (   ) del suelo. El participante se coloca de pie y de espaldas a la pared con la parte media de la cabeza sobre la cinta métrica y los ojos mirando al frente a continuación colocaremos una regla o algo similar en cima de su cabeza. La altura del participante será la puntuación indicada en al cinta métrica más las 20 pulgadas (   ) distancia desde la cinta métrica al suelo. *Ajuste*: si el participante lleva puestos los zapatos se restará a la medición entre 2 y 4 cm según el juicio del examinador.
3. Respecto al ***Peso***: el participante se quitará la ropa de mayor peso (chaqueta, jersey...) y se colocará sobre la báscula. *Ajuste*: Si pesamos al participante con los zapatos puestos se restará medio kilo si el calzado es ligero y un kilo si es un calzado pesado (siguiendo el juicio del examinador)

Puntuación:
Se anotarán los valores de peso y talla en la hoja de registro y se calculará el Índice de Masa Corporal según la siguiente fórmula:

$$IMC= peso\ (Kg.)/\ talla^2\ (m)$$

Valores de referencia

Dentro de la SFT, los *valores de referencia* nos permiten interpretar los resultados de la batería así como motivar al participante ya que una vez que han finalizado el test muchos quieren saber que puntuación han obtenido, que significado tiene esa puntuación y que pueden hacer para mejorarlo, y por supuesto nos servirá para mejorar su capacidad funcional.

Las tablas normativas y los criterios de referencia fueron desarrollados para la SFT basándose en un estudio nacional realizado a más de 7.000 mayores independientes de entre 60 a 94 años de edad, de 267 diferentes lugares de Estados Unidos.

A continuación se muestran el intervalo normal (entre el 25th percentil y el 75th percentil) según el género y en las distintas edades (desde los 60 a los 94 años de edad).

INTERVALO NORMAL EN MUJERES

	60-64	65-69	70-74	75-79	80-84	85-89	90-94
Sentarse y Levantarse de una silla (nºrep)	12-17	11-16	10-15	10-15	9-14	8-13	4-11
Flexiones de brazo (nºrep)	13-19	12-18	12-17	11-17	10-16	10-15	8-13
Caminar 6 minutos	545-660	500-635	480-615	435-585	385-540	340-510	275-440

(yardas)							
2 minutos marcha (pasos)	75-107	73-107	68-101	68-100	60-90	55-85	44-72
Flexión del tronco en silla (pulgadas)	(-0.5)-(+5.0)	(-0.5)-(+4.5)	(-1.0)-(+4.0)	(-1.5)-(+3.5)	(-2.0)-(+3.0)	(-2.5)-(+2.5)	(-4.5)-(+1.0)
Juntar las manos tras la espalda (pulgadas)	(-3.0)-(+1.5)	(-3.5)-(+1.5)	(-4.0)-(+1.0)	(-5.0)-(+0.5)	(-5.5)-(+0.0)	(-7.0)-(-1.0)	(-8,0)-(-1.0)
Levantarse caminar y volverse a sentar (seg.)	6.0-4.4	6.4-4.8	7.1-4.9	7.4-5.2	8.7-5.7	9.6-6.2	11.5-7.3

INTERVALO NORMAL EN HOMBRES

	60-64	65-69	70-74	75-79	80-84	85-89	90-94
Sentarse y Levantarse de una silla (nºrep)	14-19	12-18	12-17	11-17	10-15	8-14	7-12
Flexiones de brazo (nºrep)	16-22	15-21	14-21	13-19	13-19	11-17	10-14
Caminar 6 minutos (yardas)	610-735	560-700	545-680	470-640	445-605	380-570	305-500
2 minutos marcha (pasos)	87-115	86-116	80-110	73-109	71-103	59-91	52-86
Flexión del tronco en silla (pulgadas)	(-2.5)-(+4.0)	(-3.0)-(+3.0)	(-3.0)-(+3.0)	(-4.0)-(+2.0)	(-5.5)-(+1.5)	(-5.5)-(+0.5)	(-6.5)-(-0.5)
Juntar las manos tras la espalda (pulgadas)	(-6.5)-(+0.0)	(-7.5)-(-1.0)	(-8.0)-(-1.0)	(-9.0)-(-2.0)	(-9.5)-(-2.0)	(-9.5)-(-3.0)	(-10,5)-(-4.0)
Levantarse caminar y volverse a sentar (seg.)	5.6-3.8	5.9-4.3	6.2-4.4	7.2-4.6	7.6-5.2	8.9-5.5	10.0-6.2
